# High Stability of the Epigenome in *Drosophila* Interspecific Hybrids

**DOI:** 10.1093/gbe/evac024

**Published:** 2022-02-10

**Authors:** Alejandra Bodelón, Marie Fablet, Philippe Veber, Cristina Vieira, Maria Pilar García Guerreiro

**Affiliations:** 1 Grup de Genòmica, Bioinformática i Biologia Evolutiva, Departament de Genètica i Microbiologia (Edifici C), Universitat Autònoma de Barcelona, Spain; 2 Laboratoire de Biométrie et Biologie Evolutive, UMR5558, Université Claude Bernard Lyon 1, Villeurbanne, France; 3 Institut universitaire de France, France

**Keywords:** epigenome, *Drosophila*, interspecific hybrids, deregulation, transposable elements, histone methylation

## Abstract

Interspecific hybridization is often seen as a genomic stress that may lead to new gene expression patterns and deregulation of transposable elements (TEs). The understanding of expression changes in hybrids compared with parental species is essential to disentangle their putative role in speciation processes. However, to date we ignore the detailed mechanisms involved in genomic deregulation in hybrids. We studied the ovarian transcriptome and epigenome of the *Drosophila buzzatii* and *Drosophila koepferae* species together with their F_1_ hybrid females. We found a trend toward underexpression of genes and TE families in hybrids. The epigenome in hybrids was highly similar to the parental epigenomes and showed intermediate histone enrichments between parental species in most cases. Differential gene expression in hybrids was often associated only with changes in H3K4me3 enrichments, whereas differential TE family expression in hybrids may be associated with changes in H3K4me3, H3K9me3, or H3K27me3 enrichments. We identified specific genes and TE families, which their differential expression in comparison with the parental species was explained by their differential chromatin mark combination enrichment. Finally, *cis–trans* compensatory regulation could also contribute in some way to the hybrid deregulation. This work provides the first study of histone content in *Drosophila* interspecific hybrids and their effect on gene and TE expression deregulation.

Significance The genomic stress caused by hybridization between different species is a cause of gene and transposable element (TE) deregulation. Histone modifications were often associated to genomic deregulation, but little is known to date about their role in *Drosophila* hybrid anomalies, and their elucidation is essential to unravel the role of these phenomena in speciation. Using transcriptomic and epigenomic analyses, we found that even though the epigenome of the parental species is highly conserved in hybrids, some changes detected in histone marks were associated to gene and TE deregulation. This work provides the first study of histone modification in *Drosophila* hybrids and its putative impact on gene a TE expression.

## Introduction

Interspecific hybridizations are a source of phenotypic variation (Soto et al. 2008; Zhu, Hu, et al. 2017), sterility (Naveira and Fontdevila 1991; Michalak and Noor 2003; Moehring et al. 2007), and genomic instability (Naveira and Fontdevila 1991; Vela et al. 2014, 2011) in hybrid offspring. These phenomena are induced by the fusion of two different parental genomes and epigenomes, which act as a genomic stressful factor in the hybrid genome (García Guerreiro 2012). This integration leads to deregulation of gene expression in hybrids, which has been described in *Drosophila* (Michalak and Noor 2003; Ranz et al. 2004; Moehring et al. 2007; Romero-Soriano et al. 2017; Gámez-Visairas et al. 2020) and other organisms (Malone and Michalak 2008; Zhu, Hu, et al. 2017). For example, the misexpression of genes involved in spermatogenesis in *Drosophila* male hybrids (Michalak and Noor 2003; Moehring et al. 2007) has been related with their sterility. Deregulation of gene expression was also observed in hybrid females of *Drosophilabuzzatii* and *Drosophilakoepferae* (Romero-Soriano et al. 2017; Gámez-Visairas et al. 2020) and in other *Drosophila* hybrids (Ranz et al. 2004). Gene expression in *Drosophila* is controlled at different levels, including epigenetic modifications (Yin et al. 2011) and *cis-* and *trans-*regulatory elements, among others. Divergence between parental regulatory elements may play an important role in gene expression deregulation in hybrids (Mack and Nachman 2017). For example, differences in the *cis* and *trans* parental regulatory elements were related with misexpression in *Drosophila* hybrids (Landry et al. 2005).

In addition to gene expression disruption, a growing number of evidence in *Drosophila* suggest that TEs could also play an important role in the hybrid anomalies. For example, increases of transposition rates and/or misexpression were observed in interspecific hybrids between *D. buzzatii* and *D. koepferae* (Labrador et al. 1999; Vela et al. 2014; García Guerreiro 2015; Romero-Soriano and Garcia Guerreiro 2016) and other *Drosophila* species (Kelleher et al. 2012; Lopez-Maestre et al. 2017). In *Drosophila*, germline transposition is controlled at two levels: transcriptional silencing, by heterochromatinization processes, and post-transcriptional by piwi-interacting RNAs (piRNAs) involved in transposon transcript degradation. In this sense, the causes of TE release in hybrids have been the matter of recent research focused on the study of disruption of the germline mechanisms involved in TE regulation, basically those affecting piRNA production (Kelleher et al. 2012; Lopez-Maestre et al. 2017; Romero-Soriano et al. 2017). Recent discoveries pointed to a dysfunction of piRNA pathway in *Drosophilamelanogaster–Drosophilasimulans* hybrids (Kelleher et al. 2012), which would have a deficient global piRNA production, whereas a similar or higher piRNA production was observed in *D. buzzatii-D. koeferae* ovaries (Romero-Soriano et al. 2017). Moreover, in the later study a high proportion of the TEs overexpressed in hybrids did not have associated piRNAs in parents or hybrids, which suggests a more complex deregulation network, at least, in these hybrids.

Less is known about the behavior of epigenomes in interspecific *Drosophila* hybrids, which is of fundamental interest in evolutionary biology. Histone modification is a post-translational chemical modification of histone proteins. In *Drosophila*, the histone marks H3K4me3 are associated to activation of gene expression, whereas H3K9me3 and H3K27me3 are associated to gene repression (Yin et al. 2011). H3K4me3 marks are abundant in euchromatin, and H3K9me3 and H3K27me3 take part of constitutive and facultative heterochromatin, respectively (Ebert et al. 2006; Schulze and Wallrath 2007). Moreover, it was also shown that TEs are enriched in the H3K9me3 and H3K27me3 heterochromatic marks, suggesting a putative colocalization of these silencing marks in these sequences (Yin et al. 2011). In plants, histone modification occurring during interspecific hybridization has been associated to gene expression variation (He et al. 2010; Zhu, Hu, et al. 2017). However, in *Drosophila*, little is known about the role of histone modification on gene expression and their consequences on hybrid incompatibility and TE deregulation. Studies on *D. melanogaster–D. simulans* hybrids suggest that adaptive divergence of heterochromatin proteins is an important force driving the evolution of genes involved in hybrid incompatibility (Satyaki et al. 2014). Our previous work on *D. buzzatii–D. koepferae* hybrids suggested the existence of interacting phenomena, including incompatibilities of the piRNA pathway, due to a functional divergence between parental species, as one of the causes responsible of TE mobilization (Romero-Soriano et al. 2017; review Fontdevila 2019). However, we cannot disregard other putative mechanisms in these species, including histone modifications causing changes in TE expression.

To unravel the causes of this hybrid incompatibility, we investigated the expression profiles and the patterns of three histone marks: H3K4me3, H3K9me3, and H3K27me3, using RNA-sequencing (RNAseq) and chromatin immunoprecipitation and deep sequencing (ChIPseq) in the genome of the parental species (*D. buzzatii* and *D. koepferae*) and their hybrids. *Drosophilabuzzatii* and *D. koepferae* are two cactophilic sibling species, belonging to *repleta* group (García Guerreiro 2014), that diverged approximately 4–5 Ma (Gómez and Hasson 2003; Oliveira et al. 2012; Romero-Soriano et al. 2017). Crosses between *D. buzzatii* males and *D. koepferae* females result in F_1_ sterile males and fertile females (Marin et al. 1993), even though a few cases of partial fertility with atrophy in one of the ovaries is also observed (Marin and Fontdevila 1998). Hybrid females can be backcrossed with *D. buzzatii* males (Naveira et al. 1986; Marin and Fontdevila 1998). In this system, we can only obtain hybrids from crosses between *D. koepferae* females and *D. buzzatii* males, the reciprocal cross does not produce offspring (Marin et al. 1993).

We found that both genes and TE families detected as differentially expressed in hybrid ovaries in comparison with *D. buzzatii* and *D. koepferae*, tended to be mostly underexpressed. In contrast, we found a high conservation of the parental chromatin mark patterns in hybrid genes and TEs, showing intermediate levels between the parental species. Nevertheless, we could associate some changes in gene and TE family expression in hybrids with their corresponding histone modifications versus parental species.

## Results

### Deregulation of Gene and TE Expression in Hybrids

**Fig. 1.**
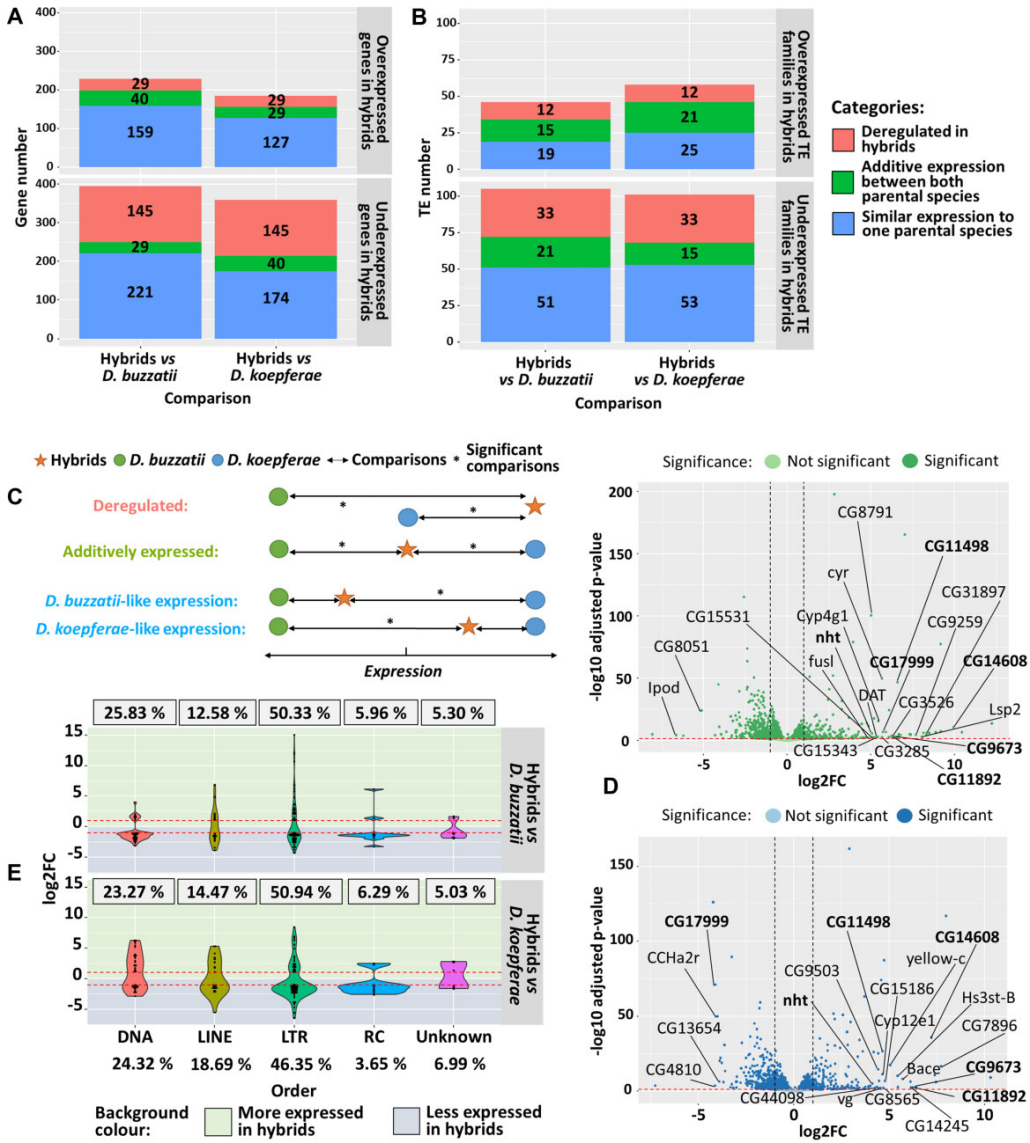
Differential expression analyses of genes and TEs: (*A*) Number of differentially expressed genes and (*B*) TE families in hybrids versus parental species of the total of 13,621 genes and 658 TE families. Colors indicate gene expression categories. (*C*) Expression categories in hybrids versus parental species. (*D*) Differential gene expression analyses in hybrids versus *D. buzzatii* (green) and *D. koepferae* (blue). Positive log2FC values correspond to genes more expressed in hybrids. The genes showing the 20 highest log2FC values and displaying an ortholog in *D. melanogaster* are shown. Genes common to both comparisons are in bold. (*E*) Violin plots representing the distribution of differentially expressed orders of TEs in hybrids versus parental species. Points indicate the log2FC of each TE family and for each comparison. The percentages of differentially expressed TE families per order are framed. The expected (total) TE family percentages are at the bottom. Red-dashed lines indicate the log2FC threshold for significancy (±1).

We analyzed and compared the ovarian transcript amounts of *D. buzzatii* and *D. koepferae* and their F_1_ female hybrids. We found that, out of a total of 13,621 protein coding genes and 658 TE families, 5.92% and 29.64%, respectively, were differentially expressed between the parental species (supplementary file 1: table S1, Supplementary Material online). Similarly, hybrids also showed a lower percentage of differentially expressed genes, in comparison with the parental species (4.57% vs. *D. buzzatii* and 3.99% vs. *D. koepferae*, fig. 1*A*), than TE families (22.95% vs. *D. buzzatii* and 24.16% vs. *D. koepferae*, fig. 1*B*). Gene expression in hybrids was more similar to the maternal species *D. koepferae* than to the paternal species *D. buzzatii* (*Z*-test, *P =* 0.018, supplementary file 1: table S2, Supplementary Material online), but the number of differentially expressed TE families in hybrids was similar in comparison with both parental species (*Z*-test, *P =* 0.603, supplementary file 1: table S2, Supplementary Material online).

Globally, we could classify the differentially expressed genes and TEs in hybrids in three categories (fig. 1*C*): 1) *D. koepferae*- or *D. buzzatii*-like expression, which corresponded to the majority of differentially expressed genes (fig. 1*A*) and TE families (fig. 1*B*). 2) Overexpressed or underexpressed in hybrids in comparison with both parental species, which were considered as deregulated in hybrids (fig. 1*C*). We observed a deregulation trend toward underexpression in hybrids compared with both parental species: 145 genes and 33 TE families underexpressed versus 29 genes and 12 TE families overexpressed (fig. 1*A* and *B*). 3) Additive expression which included differentially expressed genes and TE families in hybrids with an intermediate expression between both parental species. Only a small number of genes (29 and 40 genes) were included in this category (fig. 1*A*). Regarding the TEs, the additive expressed TE families were mostly underexpressed in comparison with *D. buzzatii*, and overexpressed in comparison with *D. koepferae* (fig. 1*B*). Both results were consistent with the differences between the parental species, where the mean TE family expression was higher in *D. buzzatii* than in *D. koepferae* (mean logarithmic 2-fold change [log2FC] = 0.27 or 1.21-fold increase in *D. buzzatii* vs. *D. koepferae*), and opposite for the genes (mean log2FC= −0.23 or 0.85-fold decrease in *D. buzzatii* vs. *D. koepferae*; supplementary file 1: table S1, Supplementary Material online).

We found that, even though there was a general bias toward underexpression of genes (fig. 1*D*) and TE families (fig. 1*E*) in hybrids, those detected as overexpressed exhibit the highest differences of expression compared with parental species. In fact, the values of mean log2FC were 2.53–3.01 (FC = 5.78–8.06) for genes and 3.33–3.56 (FC = 10.06–11.79) for TE families overexpressed in hybrids versus *D. koepferae* and *D. buzzatii*, respectively, whereas they decreased to −1.62–1.72 (FC = 0.33–0.30) for genes and to −1.85–1.74 (FC = 0.28–0.30) for TE families underexpressed in hybrids versus *D. koepferae* and *D. buzzatii*, respectively. Regarding the genes, most of the overexpressed shown in figure 1*D*, were involved in metabolism (small molecules or protein), development, signaling and stimulus response, reproduction and transportation, and cell organization (supplementary file 2, Supplementary Material online).

An interesting example was the *no hitter gene* (nht), highlighted in bold in figure 1*D*, which was overexpressed in hybrids compared with both parental species. This gene is involved in spermatogenesis and regulation of gene expression and may be related to female hybrid sterility (Ranz et al. 2004). Moreover, as expected, most of the genes with higher differences between hybrids and the parental species (gene names labeled in fig. 1*D*) were at the same time detected as the most different between the parental species (green and blue gene names in supplementary file 3: fig. S1*a*, Supplementary Material online).

Regarding underexpressed genes, the Gene Ontology (GO) analysis revealed a shared enrichment for 11 terms in hybrids versus both parental species (green GO in supplementary file 2, Supplementary Material online) and were mainly related to developmental processes, cell adhesion and reproduction (i.e., gonad development). Furthermore, the fact that most of them had functions related with reproduction, reinforces the idea of a putative role in hybrid fertility loss (Moehring et al. 2007). In the case of the overexpressed genes in hybrids, only two enriched GO terms were shared with both parental species (green GO in supplementary file 2, Supplementary Material online), and were linked to metabolic and cellular process. Additional GO terms, related to the same or other processes (such as response to stimulus and biological regulation, among others) were enriched in the differentially expressed genes, but only in comparison with one of the parental species (supplementary file 2, Supplementary Material online).

To detect a putative location bias of the differentially expressed genes in hybrids in comparison with the parental species, previously reported in some *Drosophila* hybrids (Moehring et al. 2007; Wurmser et al. 2011), we studied their distribution per chromosome. We found that the number of differentially expressed genes across chromosomes in hybrids compared with both parental species (supplementary file 3: fig. S2*a*, Supplementary Material online), was different from the random expectation (chi-square test, *P* < 0.001, supplementary file 1: table S3, Supplementary Material online). This result is due to a higher number of differentially expressed genes on the dot chromosome 6 (1.93% vs. *D. buzzatii* and 2.21% vs. *D. koepferae*) than the expected (0.54%). When this chromosome was excluded from the test, the differences become non-significant (chi-square test, *D. buzzatii*: *P* = 0.320 and *D. koepferae*: *P* = 0.443, supplementary file 1: table S3, Supplementary Material online).

Concerning the TEs, we studied the distribution and expression of the differentially expressed TE families, per order, in hybrids in comparison with the parental species. We used the Repbase classification (Jurka et al. 2005) where TEs were divided in LTR, LINE, and DNA orders. Due to their particular replication mechanism, we considered the RC/Helitron as a group apart from the DNA order. As shown in figure 1*E*, the number of differentially expressed TE families by order was similar to the random expectation considering their relative proportions in the genome (chi-square test, *P* = 0.183 for *D. buzzatii* and *D. koepferae*, supplementary file 1: table S3, Supplementary Material online). The most extreme underexpression values were observed in LINE and LTR families in hybrids versus *D. koepferae*. In contrast, these families together with RC were the most overexpressed in hybrids versus *D. buzzatii*. Last, the LTR order was the one where some families showed extreme overexpression and underexpression values in hybrids versus both parental species. When we went deeper into the superfamilies of elements belonging to each order (supplementary file 3: fig. S2*b*, Supplementary Material online), highly expressed elements in hybrids included mainly Gypsy (LTR), Helitron (RC), and Jockey (LINE) elements in comparison with *D. buzzatii* and, Gypsy (LTR), hAT (DNA), and TC1Mariner (DNA) elements compared with *D. koepferae*. Some of these TEs were detected as the most differentially expressed between the parental species (green and blue TE names in supplementary file 3: fig. S1*b*, Supplementary Material online). Consistently with what was found in previous works (Romero-Soriano et al. 2017), the most highly overexpressed TEs in hybrids versus both parental species belonged to Gypsy subfamily (supplementary file 3: fig. S2*b*, Supplementary Material online).

### Epigenetic Mark Landscapes Are Conserved and Contribute to Gene and TE Expression

**Fig. 2.**
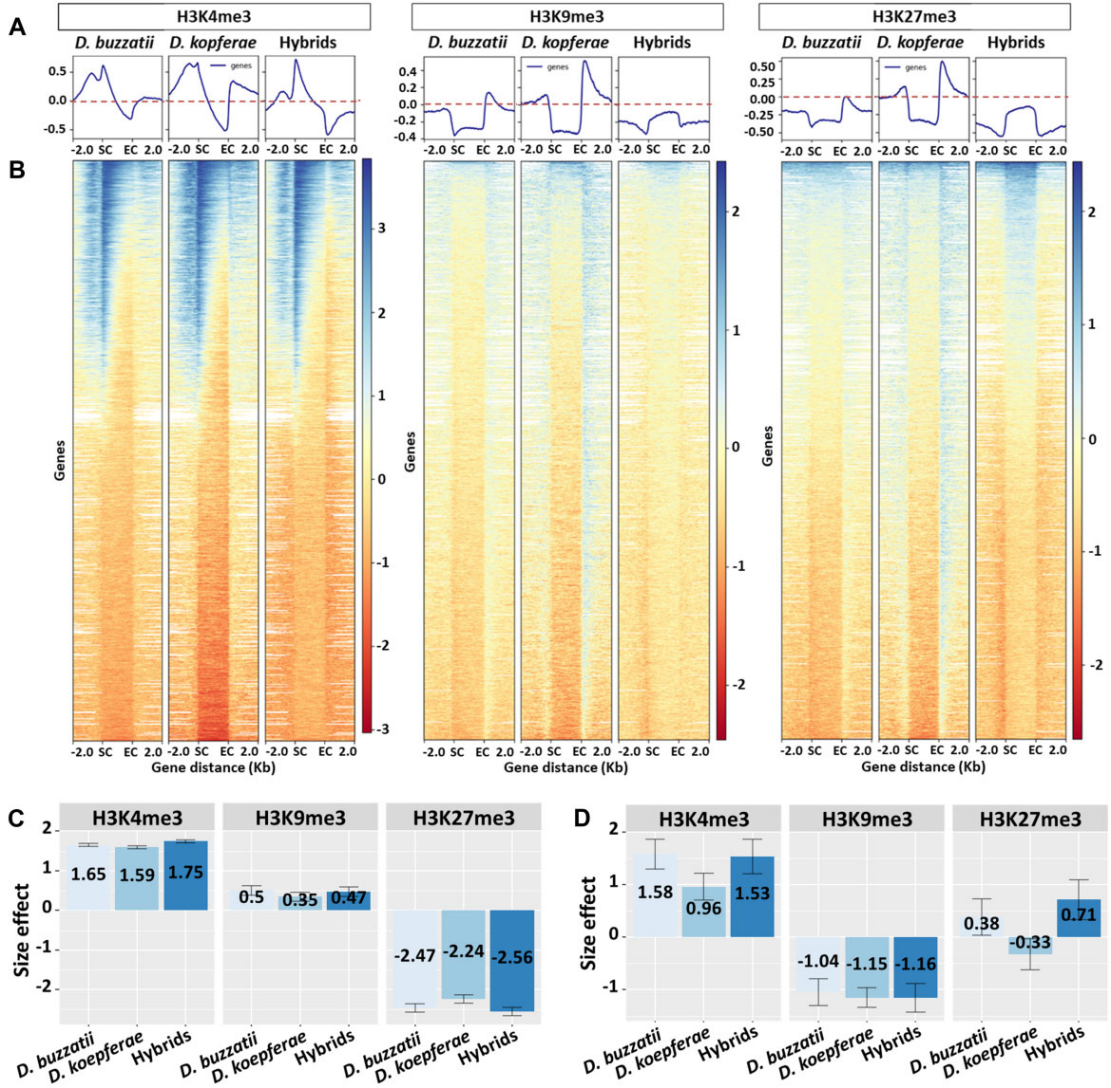
Global epigenetic patterns in *D. buzzatii*, *D.koepferae*, and hybrids and their contribution to expression: (*A*) Average levels of the histone marks H3K4me3, H3K9me3, and H3K27me3 over all annotated gene sequences (exons and introns) and the ±2 kb surrounding regions. (*B*) Heatmaps showing the density scores of each histone mark in *D. buzzatii*, *D. koepferae*, and their hybrids. White regions in the heatmaps indicate missing data. SC: start codon, EC: end codon. (*C*, *D*) Mean linear effects of H3K4me3, H3K9me3, and H3K27me3 enrichments on RNA read counts (log transformed) in (*C*) genes and (*D*) TE families. Colors indicate the species. The 95% confidence intervals are indicated.

In *Drosophila*, the constitutive heterochromatin is enriched in H3K9me3 epigenetic mark, whereas H3K27me3 and H3K4me3 are associated to facultative heterochromatin and euchromatin, respectively (Boros 2012). We investigated the landscape of epigenetic marks in the parental species, unknown to date, and in their hybrids. Analyses of the distribution of histone marks in genes and their surrounding regions (fig. 2*A* and *B*) showed that H3K4me3 euchromatic mark is enriched around the start codon (SC) and throughout the coding sequence in hybrids and their parental species, which is similar to the well-described *D. melanogaster* species. H3K9me3 and H3K27me3 epigenetic marks were mainly depleted in the gene body in all species (fig. 2*A* and *B*).

We then studied the contribution of these histone marks to the gene and TE family expression in the parental species *D. buzzatii*, *D. koepferae*, and their hybrids, using the linear model (RNA ∼ K4 + K9 + K27 + Input) described in Material and Methods. We found that 62% ($r^{2}$ adjusted) of gene expression variation was explained by the additive linear relationship with log-transformed histone mark enrichments, with a *P*-value lower than 2.2 × 10^−16^ in all species (supplementary file 1: table S4, Supplementary Material online). In the case of TE expression, the adjusted $r^{2}$ reached values from 61% in hybrids, to 75% in *D. koepferae* (supplementary file 1: table S4, Supplementary Material online).

Globally, we detected that the size effect, representing the contribution of each chromatin mark to the gene expression according to our model, was similar in hybrids and parental species (fig. 2*C*), being H3K27me3 the chromatin mark with the greatest contribution to gene expression. H3K4me3 was strongly positively associated with gene expression showing coefficient values from 1.59 in *D. koepferae* to 1.75 in hybrids, whereas H3K27me3 was strongly negatively associated: from −2.24 in *D. koepferae*, to −2.56 in hybrids (supplementary file 1: table S4, Supplementary Material online). H3K9me3 was the one with the least positive contribution to gene expression (from 0.35 in *D. koepferae* to 0.5 in *D. buzzatii*).

Regarding TEs, the contribution of H3K4me3 and H3K9me3 to the expression was similar in hybrids and parental species (fig. 2*D*). H3K4me3 was positively associated with TE expression, with coefficient values from 0.96 in *D. koepferae* to 1.58 in *D. buzzatii* (supplementary file 1: table S4, Supplementary Material online), whereas H3K9me3 was negatively associated (−1.04 in *D. buzzatii* to −1.16 in hybrids), as expected. Contrary to the pattern observed in genes, H3K27me3 was the chromatin mark with the lowest contribution to TE expression and its contribution depended on the species: positively associated in *D. buzzatii* and hybrids (0.38 and 0.71 respectively) and negatively in *D. kopferae* (−0.33).

### Maintenance of the Parental Enrichment of Epigenetic Marks in the Hybrid Genome

**Fig. 3.**
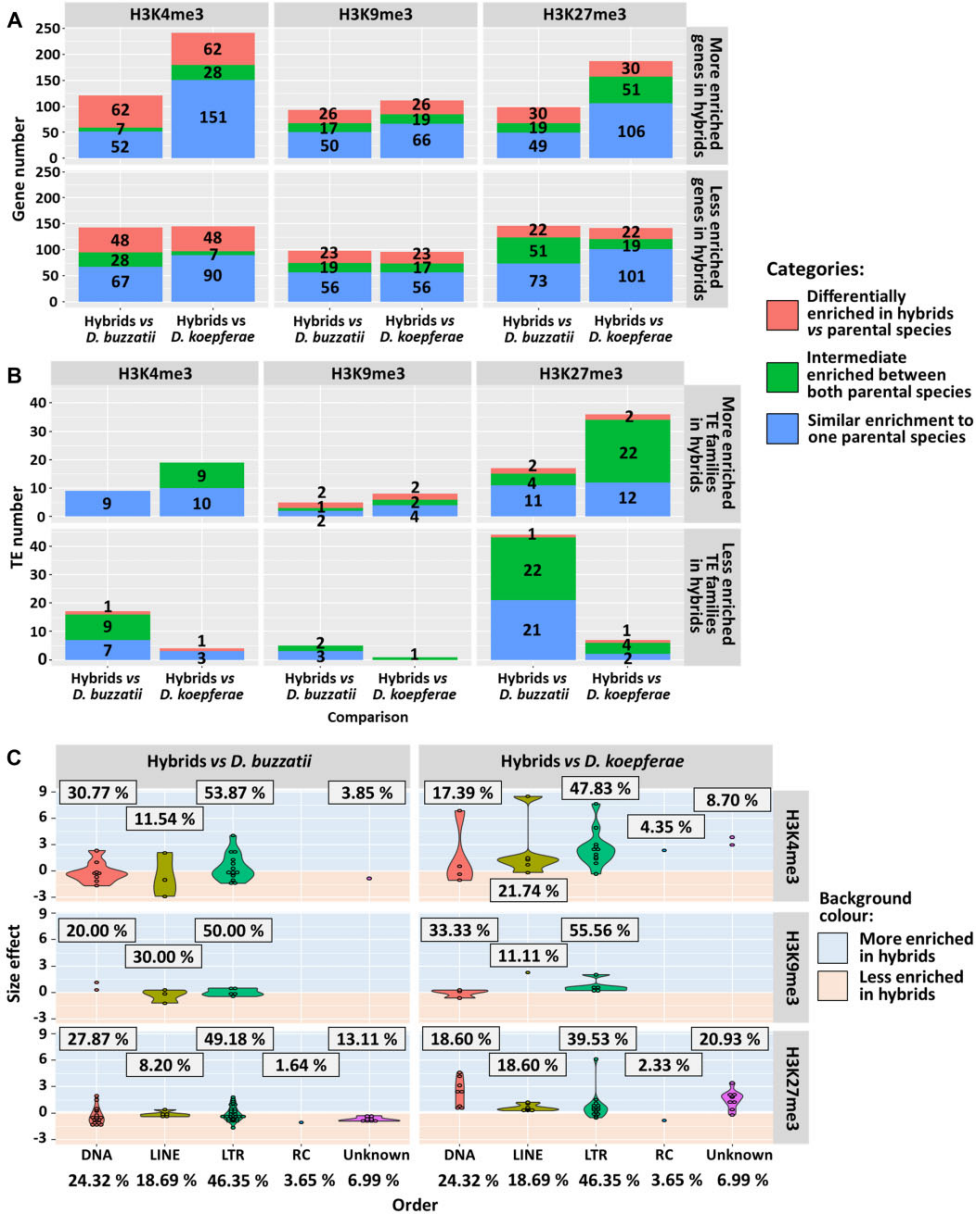
Comparison of epigenetic marks in hybrids and parental species: (*A*) Number of differentially enriched genes and (*B*) TEs of the total of 13,621 genes and 658 TE families in H3K4me3, H3K9me3, or H3K27me3 chromatin marks in hybrids versus the parental species *D. buzzatii* and *D. koepferae*, respectively. Colors indicate the different categories of enrichment. (*C*) Violin plots representing the distribution of differentially H3K4me3, H3K9me3, and H3K27me3 enriched TE orders in hybrids versus parental species. Points indicate the size effect. The percentages of differentially enriched TE families per order are framed. The expected (total) TE family percentages are at the bottom.

We computed read counts corresponding to enrichments in H3K4me3, H3K9me3, and H3K27me3, in the gene bodies and TE families from hybrids and their parental species. We found that only a small percentage of all genes (1.40–2.83%) were differentially enriched in hybrids compared with any parental species and chromatin mark (fig. 3*A*). A lower number of differentially H3K4me3 and H3K27me3 enriched genes was detected in hybrids when they were compared with *D. buzzattii* than to *D. koepferae* (*Z*-test, *P* < 0.001, supplementary file 1: table S5, Supplementary Material online). Regarding TE families, the hybrid versus parents comparisons revealed very similar patterns for H3K9me3 enrichments (1.52–1.37% of differentially enriched TE families), whereas they were the most contrasted for H3K27me3 enrichments (9.27–6.53% differentially enriched TE families, fig. 3*B*). However, the number of differentially enriched TEs in hybrids was similar to one or another parental species (*Z*-test, *P =* 0.817 H3K4me3 and H3K9me3; *P =* 0.198 H3K27me3; supplementary file 1: table S5, Supplementary Material online). Moreover, H3K9me3 was the chromatin mark with more similar patterns between hybrids and the parental species for both genes and TE families, which was consistent with the similar patterns of this chromatin mark when the parental species were compared (supplementary file 1: table S6, Supplementary Material online). On the contrary, in TE families H3K27me3 was the mark with the largest differential enrichment in hybrids versus the parental species, showing the largest contrast between the parental species (supplementary file 1: table S6, Supplementary Material online).

In general, most of the differentially enriched genes and TEs in hybrids have *D. buzzatii* or *D. koepferae-*like chromatin mark levels. However, we also observe a high number of genes displaying histone mark enrichments outside of the range of parental values, especially for H3K4me3—48 less enriched and 62 more enriched in hybrids versus both parental species (fig. 3*A*). In the same way, a high number of TE families show intermediate patterns of enrichment between the parental species (fig. 3*B*).

We studied the distribution across chromosomes of the differentially enriched genes for each epigenetic mark in hybrids in comparison with each parental species and we did not find significant differences in any case (chi-square test, *P* > 0.05 in all cases, supplementary file 1: table S7, Supplementary Material online). Globally, we observed that the extreme changes in chromatin mark levels were toward an increase in hybrids in comparison with the parental species (supplementary file 3: fig. S3, Supplementary Material online). For example, some extreme changes of the euchromatic mark H3K4me3 were observed in chromosome 5 in comparison with *D. koepferae*, and of the euchromatic mark H3K27me3 in comparison with *D. koepferae* (chromosomes 4 and 5) and *D. buzzatii* (chromosome 2).

When we studied the orders of the differentially enriched TE families in hybrids versus parental species, we did not find differences (chi-square test, *P* > 0.05 in all cases, supplementary file 1: table S7, Supplementary Material online) for any chromatin mark (fig. 3*C*). If we focus on the specific enrichment of epigenetic marks, three TEs belonging to DNA, LINE, and LTR families showed the most extreme values of H3K4me3 in hybrids versus *D. koepferae* (ranging from 6 to 9). However, RC and unknown TEs showed values of this chromatin mark similar to both parental species, being only a small percentage differentially enriched. We also found a high increment of H3K27me3 in one LTR element in comparison with *D. koepferae* (∼6), whereas the changes were less extreme in comparison with *D. buzzatii*. As reported for gene enrichment, only small differences of H3K9me3 amounts were observed in hybrids versus parental species.

### Small Changes in the Hybrid Epigenome Affect Their Gene and TE Expression

**Fig. 4.**
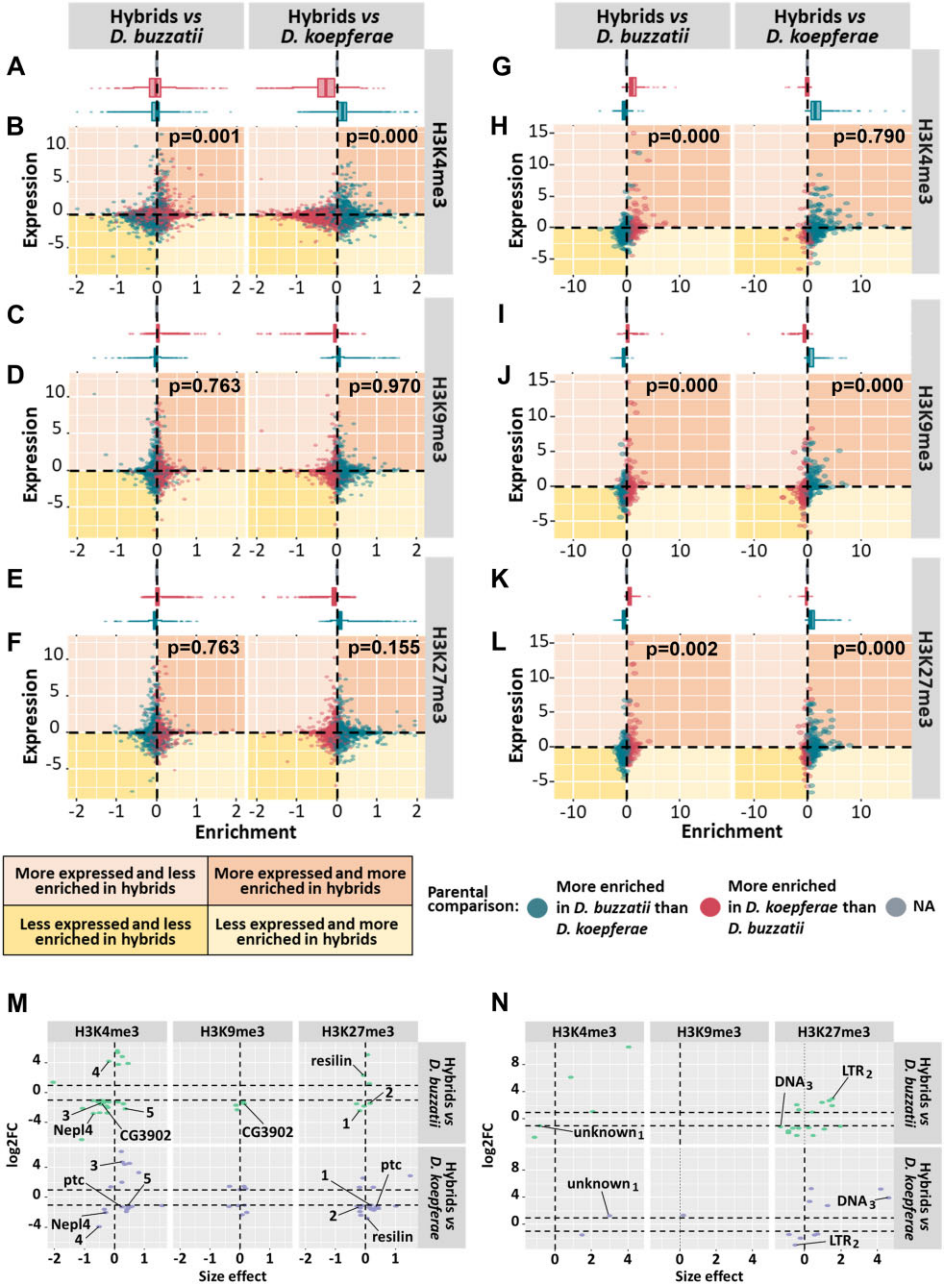
Association of expression changes with the corresponding chromatin mark changes: (*A–L*) Differential expression (log2FC) and differential enrichment (size effect) of H3K4me3, H3K9me3, and H3K27me3 in all genes (*B*, *D*, *F*) and TE families (*H*, *J*, *L*) (significant and nonsignificant) in hybrids versus *D. buzzatii* and *D. koepferae*, respectively. Top plots represent the distribution of the differential enrichment between parental species in genes (*A*, *C*, *E*) and TE families (*G*, *I*, *K*). Colors represent the results of the differential enrichment between parental species, and values that cannot be computed are included in the NA category. Fisher’s exact test *P*-values are shown in the right corner. Gene outliers are not included in (*A–F*). (*M*, *N*) Log2FC and size effect representation of significant differentially expressed and enriched genes (*M*) and TEs (*N*) in hybrids versus the parental species. Significant genes (*M*) in at least two comparisons are named using their *D. melanogaster* ortholog or, otherwise, a number. The TE families (*N*) common to at least two comparisons are marked, indicating their category and a subscript.

We analyzed the association between gene and TE expression changes in hybrids in comparison with the parental species and the corresponding changes in chromatin marks (fig. 4*A*–*L*, and supplementary file 1: table S8, Supplementary Material online) using the whole set of genes and TE families (significant and nonsignificant). Regarding the genes, we found that changes in the enrichment of the heterochromatic marks H3K9me3 and H3K27me3 were not associated with expression changes in hybrids in comparison with the parental species (Fisher’s exact test, *D. buzzatii*: *P = 0.763* both chromatin marks; *D. koepferae*: *P = 0.970* and *P = 0.155*, respectively, supplementary file 1: table S8, Supplementary Material online). However, changes in H3K4me3 in hybrids seemed to be associated with gene expression changes (Fisher’s exact test, *P ≤* 0.001: comparison with both parental species, supplementary file 1: table S8, Supplementary Material online). Indeed, genes that were underexpressed in hybrids compared with *D. buzzatii* also showed a reduced H3K4me3 enrichment. However, and unexpectedly, genes that were underexpressed in hybrids compared with *D. koepferae* also displayed an increased H3K4me3 enrichment. This apparent inconsistency could be explained by the differences in enrichment of this epigenetic mark between parental species: the genes enriched in H3K4me3 in hybrids versus *D. koepferae* often corresponded to those more enriched in *D. buzzatii* than in *D. kopferae* (blue color, fig. 4*A*), the opposite was also observed.

TE families, as genes, showed an intermediate inheritance of the chromatin marks in hybrids: TEs more enriched in a chromatin mark in hybrids in comparison with one parental species tended to be less enriched when they were compared with the other parental species (fig. 4*G*, *I* and *K*). In addition, we found an association between TE expression changes in hybrids in comparison with the parental species and the corresponding changes in the epigenome. For example, an impoverishment of the heterochromatic mark H3K9me3 was found in many underexpressed TEs in hybrids compared with both parental species (Fisher’s exact test, *P* < 0.001, supplementary file 1: table S8, Supplementary Material online). In the same way, a high TE number showing a decrease of H3K4me3 or H3K27me3 chromatin marks were underexpresed in hybrids in comparison with *D. buzzatii* (Fisher’s exact test, H3K4me3: *P* < 0.001 and H3K27me3: *P =* 0.002, supplementary file 1: table S8, Supplementary Material online). These results were opposite to those found in hybrids compared with *D. koepferae*, where an increase of H3K27me3 was observed in underexpressed elements (*P* < 0.001, supplementary file 1: table S8, Supplementary Material online). To determine if there was an association between chromatin marks in TEs, we checked the correlation between the euchromatic mark H3K4me3 and the other two, in hybrids in comparison with the parental species (supplementary file 3: fig. S4, Supplementary Material online). We found that the increase of H3K4me3 in hybrids was associated with an increase of H3K9me3 and H3K27me3 (*Linear model*, *P ≤* 1.669 × 10^−15^ in all cases, supplementary file 1: table S9, Supplementary Material online) and the opposite, with some TEs highly expressed and extremely enriched in H3K4me3 in hybrids (red dots in the upper part right of supplementary file 3: fig. S4, Supplementary Material online). When we focus on the genes considered as differentially expressed and differentially enriched in hybrids, we observe that the epigenetic mark associated with the most extreme expression changes in hybrids (absolute log2FC values ranging from 4 to 6) in comparison with both parental species was H3K4me3 (fig. 4*M*). Changes in the remaining chromatin marks were related with less extreme expression changes (absolute log2FC values from 1 to 3). Regarding the TEs showing significant differences in enrichment and expression in hybrids versus parental species (fig. 4*N*), we observed two TE families whose increase in H3K4me3 was associated to an extreme overexpression in hybrids in comparison with *D. buzzatii* (log2FC from 6 to 11). Additionally, increases in the number of H3K27me3 marks in hybrids in comparison with *D. kopferae* were likely related to extreme overexpression in hybrids (log2FC from 3 to 6). Chromatin mark changes in the remaining TEs were related with less extreme expression changes (absolute log2FC values from 1 to 3).

If we focus on the expression and chromatin enrichment of specific genes (fig. 4*M*) in hybrids, we observe some examples whose expression is explained by the combination of different chromatin marks. For example, the *CG3902* gene, involved in oxidation–reduction processes, was underexpressed in hybrids in comparison with *D. buzzatii* and less enriched in H3K4me3 but more in H3K9me3. The gene *ptc*, involved in different processes including gonad development, was underexpressed in hybrids in comparison with *D. koepferae* and more enriched in H3K4me3 and H3K27me3. In other cases, the expression changes in genes (fig. 4*M*) and TEs (fig. 4*N*) could be explained by the content of a unique epigenetic mark when hybrids are compared with one or another parental species. For example, the gene *Nepl4* (fig. 4*M*), involved in protein processing, had a low H3K4me3 content in hybrids versus both parental species and was underexpressed in hybrids. On the other hand, the gene named *3* (fig. 4*M*) and the unknown_1_ TE (fig. 4*N*) were detected as underexpressed and less enriched in H3K4me3 in hybrids in comparison with *D. buzzatii*, but overexpressed and more enriched in this chromatin mark in comparison with *D. koepferae*. Finally, LTR_2_ and DNA_3_ were enriched in H3K27me3 in comparison with one parental species, but the opposite in comparison with the other (fig. 4*N*).

### Additional Mechanisms Influencing Hybrid Deregulation

**Fig. 5.**
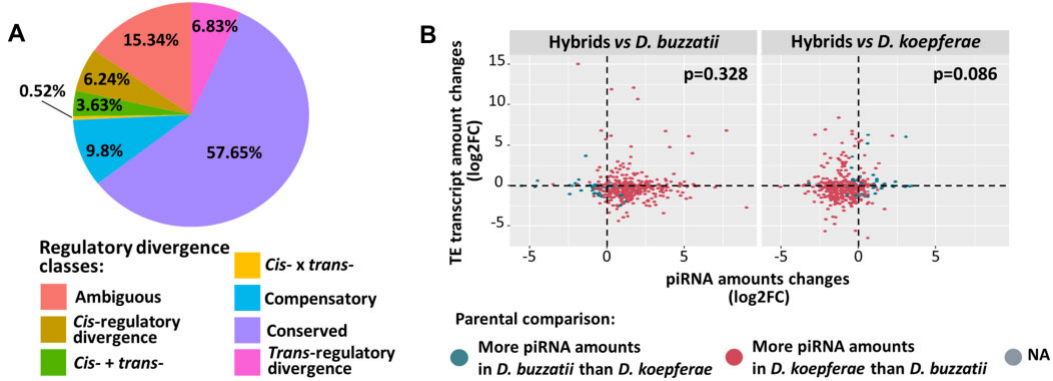
Additional factors affecting gene and TE expression, respectively: (*A*) *cis-* and *trans-* regulatory divergence between parental species. The pie chart represents the percentage of total genes categorized by regulatory divergence class. (*B*) Differential expression (log2FC) values of TEs and piRNA amounts (log2FC) in hybrids versus *D. buzzatii* and *D. koepferae*, respectively. Colors represent the results of the piRNA amount changes between parental species. Values that cannot be computed are included in the NA category. Fisher’s exact test *P*-values are shown in the right corner.

The divergence in *cis-* and *trans-* regulatory elements was proposed as one of the causes of expression differences between *Drosophila* species, as well as of gene deregulation in their hybrids (Wittkopp et al. 2004; Landry et al. 2005; McManus et al. 2010). To detect regulatory divergence between *D. buzzatii* and *D. koepferae* and its putative effects on hybrid expression, we compared the relative allele expression in hybrids (H), the differential expression between parental species (P), and the ratio between these two metrics (T). We categorized the genes in different classes, as in (McManus et al. 2010) and described in Material and Methods. As shown in figure 5*A*, most of the genes (57.65%), categorized as conserved, do not have regulatory divergence between parental species. However, we found slightly more genes showing evidence of *trans-*regulatory divergence (6.83%) than *cis-*regulatory divergence (6.24%) (*Z*-test, *P =* 0.049, supplementary file 1: table S10, Supplementary Material online). We also studied how the regulatory divergence influences expression differences between parental species. We found more differentially expressed genes between parental species with *trans-*regulatory divergence (Fisher’s exact test, *P* < 0.01, supplementary file 1: table S11, Supplementary Material online) than expected, whereas no differences to what was expected were observed in genes with *cis-*regulatory divergence (Fisher’s exact test, *P* = 0.128 for *cis-*regulatory, supplementary file 1: table S11, Supplementary Material online). Finally, we studied how the regulatory divergence observed in parents influenced the inheritance of gene expression in hybrids. We analyzed genes with regulatory divergence between *D. koepferae* and *D. buzzatii*, which were differentially expressed in hybrids versus parental species, using the expression categories described previously (fig. 1*C*). We found a higher number of differentially expressed genes with *trans-*regulatory divergence than with other regulatory classes in all expression categories, except those of genes deregulated in hybrids. In this latter category, we also observed a significantly higher number of genes with compensatory regulation (*cis-* and *trans-*regulatory differences compensate each other) than in the other expression categories (additive or *D. buzzatii/koepferae*-like) (Fisher’s exact test, additive expression: *P* = 0.039, *D. buzzatii* and *koepferae*-like expression: *P* < 0.001, supplementary file 1: table S12, Supplementary Material online).

To gain insight into other mechanisms affecting TE deregulation in hybrids, we examined the expression of piRNA pathway genes considering their role in germline development, epigenetic regulation, and TE silencing. We observed that *aubergin*e (aub) and *Sister of yellow body* (SoYb) were deregulated toward underexpression in hybrids in comparison with both parental species (supplementary file 3: fig. S5, Supplementary Material online). The chromatin mark levels were similar to the parental species, with only a few genes differentially enriched, mostly in comparison with only one parental species. Globally, differentially enriched genes decreased H3K4me3 levels in hybrids in comparison with the parental species. *Yellow body* (Yb), which was detected as overexpressed in hybrids versus both parental species, was intermediate enriched in H3K27me3 in hybrids versus both parental species, whereas *vreteno* (vret), which had an additive expression in hybrids versus parental species, was less enriched in H3K4me3 in hybrids versus both parental species. *Brother of Yellow body* (BoYb) and *armitage* (armi), which were detected as overexpressed in hybrids in comparison with one parental species, were more enriched in H3K4me3 and less enriched in H3K9me3, respectively, in comparison with the same parental species. However, most differentially enriched genes were not detected as differentially expressed (supplementary file 3: fig. S5, Supplementary Material online).

We next studied the association of piRNA amounts and TE expression in hybrids and parental species. We observed that, on average, parental species showed differences in piRNA amounts: TE families were associated with more piRNAs in *D. koepferae* than in *D. buzzatii* (mean log2FC of −2.14 in *D. buzzatii* vs. *D. koepferae* comparison, fig. 5*B*), as observed in our previous work (Romero-Soriano et al. 2017). Hybrids exhibited an additive pattern of piRNA amounts between parental species: less piRNA amounts than *D. koepferae* and more than *D. buzzatii*. Additionally, as shown in figure 5*B*, the differences in piRNA amounts were not associated to the changes in TE transcript amounts in hybrids in comparison with the parental species (Fisher’s exact test, *P =* 0.328 for *D. buzzatii* and *P* = 0.086 for *D. koepferae*, supplementary file 1: table S13, Supplementary Material online). There were also no differences when TE classes were analyzed separately (Fisher’s exact test, *P =* 0.732 for Class I and *P* = 0.564 for Class II, supplementary file 1: table S13, Supplementary Material online).

## Discussion

### Bias to Underexpression of Genes and TEs in Hybrid Females

The analysis of the total ovarian transcriptome of parental species showed that 5.92% and 29.64% of the protein-coding genes and TE families, respectively, were differentially expressed between *D. buzzatii* and *D. koepferae* parental species. These differences are similar to those obtained in other studies with other species of the *repleta* group (Lopez-Maestre et al. 2017). When the transcriptome of F_1_ hybrids and their parental species were compared, around ∼4% of genes and ∼23% of TEs from hybrids were differentially expressed compared with any parental species. A greater deregulation of TEs than of genes was already observed in a previous work with *D. melanogaster–D.simulans* artificial hybrids (Kelleher et al. 2012), but the percentages were different: 0.7% of genes were deregulated and 12% of TE families were overexpressed in comparison with both parental species (no data provided about underexpression). In contrast, ∼78% of differentially expressed genes of *D. melanogaster*–*D.simulans* female hybrid versus their parental species were found in other studies (Ranz et al. 2004). These discrepancies could be due to the different methodological approaches used along with the different genetic background of the *Drosophila* stocks.

On the other hand, in our hybrid females most of the differentially expressed genes tended to be underexpressed, as observed in previous studies in plants (Wang et al. 2006) and in *Drosophila* females (Ranz et al. 2004). The underexpression seems also to be the rule in *Drosophila* hybrid males between species of the *melanogaster* group (Michalak and Noor 2003; Moehring et al. 2007), which have been associated to male sterility. In our case, even if most F_1_ hybrid females are fertile, some are partially fertile (Marin and Fontdevila 1998), which could explain that GO terms of our underexpressed genes are associated to developmental processes, cell adhesion, and reproduction. Instead, only a few genes were extremely overexpressed in hybrids, for example the *nht*, which is involved in spermatogenesis processes. The overexpression of male-specific genes in female hybrids is not new, and has been attributed to a failure in the mechanisms controlling the expression of these genes in females (Ranz et al. 2004).

If we focus on the resemblance of gene expression in hybrids versus parental species, we found a bias toward genes that resemble more to one of the parental species, being the number of genes having an additive expression between both parental species low, as observed in previous studies with *D. melanogaster–D.simulans* hybrids (Ranz et al. 2004). Moreover, the number of genes in hybrids sharing similar transcript amounts with *D. koepferae* was higher than with *D. buzzatii*. Maternal effects were pointed out in *Arabidopsis lyrata* (Videvall et al. 2016), *Xenopus* (Malone and Michalak 2008), and in coral (Chan et al. 2021) intraspecific hybrids. Because only one direction of cross can be performed in our case, it is difficult to attribute these results unequivocally to maternal effects.

When the distribution of the derepressed genes in F_1_ hybrid females along chromosomes was considered, we did not find an overrepresentation of deregulated genes in any chromosome, except in chromosome 6 (dot). This bias could be explained if this chromosome was different between our parental species, which is reinforced by the highest rate of molecular evolution found in this dot chromosome in the closely related species *D. mojavensis* (Allan and Matzkin 2019). No bias was found in a previous *Drosophila* study (Michalak and Noor 2003). However, an X-chromosome bias of differentially expressed genes was found in other studies performed in *Drosophila* hybrid males (Moehring et al. 2007; Wurmser et al. 2011). The faster evolution of X-linked genes (Coyne and Orr 1989) proposed to explain the differential expression between X and autosomal genes has, however, been questioned by other authors (Hollocher and Wu 1996) who consider that the homozygous autosome effects in reproductive isolation, approach those of X chromosome.

We showed that 6.84% of TE families were completely deregulated in F_1_ hybrid females, in comparison with both parental species, and had a trend toward underexpression. These results, obtained using a different approach (normalization of TE counts using gene counts) and updated analysis tools, contrast with our previous results where the number of TE families upregulated slightly exceeded that of downregulated (Romero-Soriano et al. 2017). However, a few TE families belonging to the Gypsy superfamily showed values of expression very high compared with parental species, concordantly with previous results in these species where an increase of transposition and expression of the *Osvaldo* element was shown (Labrador et al. 1999; Vela et al. 2014; García Guerreiro 2015). Results on TE expression in hybrids reported in the literature are very heterogeneous, finding cases of overexpression of specific elements by RT-qPCR (Carnelossi et al. 2014; García Guerreiro 2015) or by RNAseq (Kelleher et al. 2012). However, examples of underexpression in hybrids, affecting most (Renaut et al. 2014) or a high percentage of TEs were also reported (Dion-Côté et al. 2014), but usually results of underexpression, if they exist, are poorly discussed. Finally, no evidence of TE reactivation was found in natural hybrid lineages of *Saccharomyces*, suggesting that other factors like population structure and hybrid genotype are major determinants of TE content (Hénault et al. 2020). The finding of underexpressed TE families in our hybrids highlights that regulation of some TEs exits in a way. Because cases of overexpressed TE families were also observed, we suggest that deregulation processes could be closely related to the TE family and the genetic background of species involved in the hybridization processes.

### The Epigenomic Landscapes of Parental Species and Hybrids Are Similar to Other *Drosophila* Species

Gene and TE deregulation in *D. buzzatii* and *koepferae* hybrids were previously described in the literature (Soto et al. 2008; Vela et al. 2014; García Guerreiro 2015; Romero-Soriano and Garcia Guerreiro 2016; Romero-Soriano et al. 2017; Gámez-Visairas et al. 2020), as well as in other hybrids of *Drosophila* (Moehring et al. 2007; Ortíz-Barrientos et al. 2007; Satyaki et al. 2014) and of other organisms (De Araujo et al. 2005; He et al. 2013; Zhu, Greaves, et al. 2017). To get insight about these genomic deregulation mechanisms, we studied the epigenomic landscape of a euchromatic mark (H3K4me3) and two heterochromatic marks (H3K9me3 and H3K27me3) in *D. buzzatii*, *D. koepferae*, and their F_1_ hybrids, and we found a high similarity across these species. Our results do not globally differ from those of the *D. melanogaster* species epigenome described in the literature and are consistent with the reported high conservation of the active chromatin epigenome landscapes across *Drosophila* species (Brown and Bachtrog 2014). As reported in other studies (modENCODE Consortium et al. 2011; Yin et al. 2011; Boros 2012; Parey et al. 2019), we found the H3K4me3 active chromatin mark located at the 5′ ends of actively transcribed genes and a depletion of H3K9me3 and H3K27me3 in the gene body. H3K9me3 was previously described to be enriched in promoters but depleted in 5′ transcribed regions of active genes (Yin et al. 2011; Boros 2012), whereas the H3K27me3 was underrepresented in the gene body (Filion et al. 2010; Yin et al. 2011; Boros 2012). However, both were reported to be enriched in transposons and repetitive sequences in *Drosophila* (Yin et al. 2011; Parey et al. 2019) and other organisms (Walter et al. 2016; Montgomery et al. 2020).

We next investigated whether the genomic expression was explained by the epigenetic marks, finding that globally the gene expression was positively correlated with the active mark and was negatively correlated with the H3K27me3 repressive mark, concordantly with previous studies in *Drosophila* (Yin et al. 2011) and plants (He et al. 2010). Correlations between the epigenomic landscape and gene expression in *Drosophila* have been previously described in other studies (Schübeler et al. 2004; Yin et al. 2011). According to our model, 62% of gene expression was explained by epigenetic marks, being H3K27me3 the one that most influence gene expression, followed by H3K4me3 as observed in other *Drosophila* studies (Yin et al. 2011). H3K4me3 was positively associated with gene expression, consistent with its enrichment in actively transcribed genes found in other works (modENCODE Consortium et al. 2011; Yin et al. 2011; Boros 2012; Parey et al. 2019). H3K27me3 was found to be negatively correlated to mRNA levels, consistent with its reported function of binding target for Polycomb repressive complex 1 (PRC1) (Riddle et al. 2011; Yin et al. 2011; Boros 2012; Parey et al. 2019). Surprisingly, the epigenetic mark H3K9me3, usually associated to heterochromatin regions (Yin et al. 2011), was positively associated with the gene expression, even if its contribution is very low. Increases of this epigenetic mark, along with other euchromatic ones, have been described in intronic and 3′ end regions of some active heterochromatic genes in some stages of *D. melanogaster* (Riddle et al. 2011; Saha et al. 2019).

The epigenetic marks found on TEs explained more than 60% of their expression, being H3K9me3 and H3K4me3 the most influential, with a negative and positive association to TE expression, respectively. H3K9me3 together with H3K27me3, are known to be abundant in TEs and are associated to their silencing (Walter et al. 2016; Parey et al. 2019) and H3K4me3 to their activation in *Drosophila* (Riddle et al. 2011; Yin et al. 2011) and other organisms (Walter et al. 2016). The different association of H3K27me3 with some species (positively correlated with TE expression in *D. buzzatii* and hybrids) could be explained by the different chromatin marks associated to each TE copy inside the same family.

### Hybrids Exhibited Limited Changes in Histone Marks

We studied differences in the enrichment of three chromatin marks (H3K4me3, H3K9me3, and H3K27me3) in genes and TEs between the parental species *D. buzzatii*, *D. koepferae*, and their hybrids, constituting a pioneer study in the *Drosophila* genus. We found that most of the genomic regions in hybrids show similar histone modification patterns compared with the parental species. This is consistent with the maintenance of the parental histone modification patterns, as well as their additive inheritance in intraspecific (Moghaddam et al. 2011; Dong et al. 2012; Zhu, Greaves, et al. 2017) and interspecific (Zhu, Hu, et al. 2017) plant hybrids described in the literature. However, we also observe that 1.40–2.83% of genes and 1.37–9.27% of TE families are differentially enriched in hybrids compared with any parental species. H3K4me3 seems to be related with most gene expression changes, consistent with the findings in rice and maize hybrids (He et al. 2010, 2013) where gene expression changes were correlated to the enrichment of this epigenetic mark.

In the case of TEs, TE family expression changes in hybrids in comparison with *D. buzzatii* were related to their corresponding enrichment changes in H3K4me3, H3K9me3, and H3K27me3, whereas they were related only to H3K9me3 and H3K27me3 enrichment changes when hybrids were compared with *D. koepferae*. These results are in disagreement with previous results reported in interspecific *Arabidopsis* hybrids, where changes in H3K9me2 and H3K27me3 do not coincide with the TEs having their expression changed (Göbel et al. 2018). These differences could be explained by the small number of differentially expressed TEs found in their hybrids and the use of a different organism and methodology. Because in our study, we cannot distinguish individual TE copies, they were analyzed at a family level (658 TE families), meaning that changes in expression or chromatin mark amounts are the result of the addition of the different copies per family. Indeed, intraspecific and interspecific variations in histone marks were observed in *Drosophila* TE copies (Rebollo et al. 2012), indicating that specific epigenetic modifications in TE individual copies in our hybrids could go unnoticed. Further inspection showed that increases of H3K4me3 were associated with increases of H3K9me3 and H3K27me3 in hybrids. Changes in the epigenetic status in individuals submitted to other stress were already observed in *Bari-Jheh* TE: a H3K9me3 dominant pattern turned to increases in H3K4me3, H3K9me3, and H3K27me3 enrichments (Guio et al. 2018).

### Other Mechanisms May Have a Role in Hybrid Genome Deregulation

Even though in hybrids we found a relationship between epigenetic status and expression changes in genes and TEs, the histone modification patterns did not account for the whole genome deregulation. To get more insight into this aspect, we studied the regulatory divergence between *D. buzzatii* and *D. koepferae*, and its contribution to gene expression differences between them and their hybrids. In general, we found that most genes did not have regulatory divergence between parental species, which is consistent with the small percentage (5.92%) of differentially expressed genes found between *D. buzzatii* and *D. koepferae*. We also detected more *trans-*regulatory than *cis-*regulatory divergence between these species, as well as a higher number of differentially expressed *trans*-regulated genes. These results are in concordance with the high *trans*-regulatory divergence reported between *D. melanogaster* and *D. sechellia* species (McManus et al. 2010) and the association of this class with gene differential expression between parental species, but opposite to other studies in *D. melanogaster* and *D. simulans* (Wittkopp et al. 2004; Graze et al. 2009). When we studied how the regulatory divergence affects the gene inheritance in hybrids, we found that most of the differentially expressed genes in hybrids versus parental species had *trans-*regulatory divergence. These results are in contrast to what was reported in the additive expressed genes, where *cis-*regulatory changes were more frequent than *trans-*regulatory changes (Lemos et al. 2008). Finally, we also found a high number of deregulated genes in hybrids with *cis–trans* compensatory evolution, which was also previously reported in *D. melanogaster* and *D. simulans* hybrids and considered an important cause of hybrid deregulation (Landry et al. 2005). The differences reported, both between previous works and our own, could be due to use of different *Drosophila* species, different methodologies and the use, in this work, of ovarian tissues versus whole adults.

We as well examined the expression of piRNA pathway genes for their role in germline TE silencing and we found that *aub* and *SoYb* were both underexpressed, whereas Yb was overexpressed in hybrids versus both parental species. Results of expression of these genes showed the same general trend as observed in a previous study with these species using a different analysis approach of RNAseq data (Romero-Soriano et al. 2017). Nevertheless, overexpression of three additional piwi pathway genes in the same hybrids was detected by RT-qPCR in our previous work (Gámez-Visairas et al. 2020), suggesting that the activation of these genes could be a primary response to the hybridization stress. Discrepancies found between RT-qPCR and RNAseq results highlight the different sensibilities of these two techniques. In addition, we found that the chromatin mark levels of the piRNA pathway genes was similar to the parental species, with only differences in a few genes, which is consistent to the general trend observed in other genes. In addition, we found that the piRNA amount changes were not associated with TE expression changes in hybrids, because we found an intermediate inheritance of piRNAs between the parental species in the hybrid, being *D. koepferae* the parental species with the highest amount, as observed in our previous work (Romero-Soriano et al. 2017).

We suggest that changes in transcript amounts in hybrids are either the result of the enrichment/impoverishment of a specific mark or the disproportion in the active/repressive mark content, together with other mechanisms such as *cis*–*trans* compensatory regulation. Three histone marks, considered as relevant to the expression in *Drosophila*, were considered in this work, but we cannot discard the effect of other epigenetic marks like H3K9ac highly positively correlated to gene expression (Yin et al. 2011). Other factor that could affect gene and TE deregulation is asynapsis, frequently observed in our hybrids and reported in other *repleta* hybrids (Naveira and Fontdevila 1991). Asynapsis, is known for influencing the *trans* regulation of *Ultrabithorax* gene alleles in *Drosophila* (Goldsborough and Kornberg 1996), for contributing to mouse intersubspecific hybrids infertility (Bhattacharyya et al. 2013) and to the female meiotic losses in mammals (Cloutier et al. 2015).

## Conclusions

The hybridization between *D. buzzatii* and *D.koepferae* species promotes a higher deregulation in TE families than in genes, both toward underexpression. The epigenome of the parental species is in general highly preserved in the hybrids, but some changes of the parental chromatin landscape are also observed in hybrids and are associated with their new gene and TE family expression patterns. Finally, *cis–trans* compensatory regulation could also be involved in expression deregulation of some genes. The present study has contributed to a better understanding of the mechanism affecting genomic deregulation in hybrids. Nevertheless, we cannot discard additional mechanisms, resulting from the incompatibility of the two different paternal genomes in the hybrids, which could interact forming a complex gene network and contribute to the deregulation patterns observed. This and the fact that this study is limited to hybrid females, makes that additional studies are necessary to go deeper into a better knowledge of the regulatory mechanisms and the factors involved in hybrid male sterility.

## Materials and Methods

### *Drosophila* Stocks and Crosses

We used *D. buzzatii* Bu 28 and *D. koepferae* Ko2 inbred strains described in our previous works (Vela et al. 2014; García Guerreiro 2015; Romero-Soriano et al. 2016; Romero-Soriano and Garcia Guerreiro 2016). Both strains were maintained by brother–sister mating for at least a decade and then by mass culturing. We performed ten different interspecific crosses of ten *D. buzzatii* males to ten *D. koepferae* females, due to the scarce offspring obtained in interspecific crosses. All stocks and crosses were reared at 25 °C in a standard *Drosophila* medium supplemented with yeast.

### Chromatin Preparation

Ovaries of 5-day-old *Drosophila* females were dissected in PBT (1× phosphate-buffered saline and 0.2% Tween 20). We performed chromatin extraction from two biological replicates of 50 ovaries per sample of parental species and 70 ovaries for hybrids. Samples were resuspended in Buffer A1 (HEPES 15 mM, sodium butyrate 10 mM, KCl 60 mM, Triton x100 0.5%, NaCl 15 mM) plus 1.8% formaldehyde, homogenized with a Dounce tissue grinder (15 times) and incubated for 10 min at room temperature. The crosslink was then stopped with glycine to a final concentration of 125 mM. Samples were subsequently incubated 3 min, kept on ice and washed three times with Buffer A1. We then added 0.2 ml of lysis buffer (HEPES 15 mM, EDTA 1 mM, EGTA 0.5 mM, sodium butyrate 10 mM, SDS 0.5%, sodium deoxycholate 0.1%, *N*-lauroylsarcosine 0.5%, Triton x100 1%, and NaCl 140 mM) and incubated 1–2 h at 4 °C. After the lysis process, we sonicated the samples using Biorruptor pico sonication device from Diagenode: 32 cycles of 30 s ON/30 s OFF, for parental species, and 35 cycles of 30 s ON/30 s OFF for hybrids. The sheared crosslinked chromatin was recovered from the pellet after a spin step at 10,000 g 4 min at 4 °C.

To check DNA size, samples were previously de-crosslinked with NaCl 5M, boiled 15 min and treated with 1 µl of 10 mg/ml RNAse A (37 °C, 30 min). They were purified with phenol-chloroform, precipitated in absolute ethanol, washed in 70% ethanol, resuspended in 20 µl of DEPC and run in a 1.5%-agarose gel.

For the immunoprecipitation step, Magna ChIPTM G chromatin immunoprecipation Kit (Millipore) was used together with antibodies against H3K4me3 (Abcam; ab8580), H3K9me3 (Abcam; ab8898), and H3K27me3 (Abcam; ab6002). We separated 20 µl of chromatin for input and the remaining 180 µl were distributed in three aliquots where 1 µl of each antibody plus 20 µl of magnetic beads and dilution buffer up to 530 µl were added. Samples were incubated overnight at 4 °C in an agitation wheel. After the beads were washed with low salt buffer, high salt buffer, LiCl buffer, and TE buffer. Separation of chromatin from the beads was performed using 0.5 ml of CHIP elution buffer. One microliter of Proteinase K was then added to each sample and incubated at 62 °C for 2 h in a shaker at 300 rpm. Samples were then purified with the columns provided by the kit and stored at −20 °C. ChIP enrichment was quantified by real-time PCR of a well-known genomic region enriched for the different histone marks studied: *rp49* for H3K4me3, *kkv* for H3K9me3, and *light* for H3K27me3. The following gene-specific primers were used: rp49-forward: 5′-GTCGTCGCTTCAAGGGCCAAT-3′, rp49-reverse: 5′-ATGGGCGATCTCACCGCAGTA-3′, kkv-forward: 5′-TAATCCAGCCACGCCCATTT-3′, kkv-reverse: 5′-CCCAACGTTTGCATTGCTGA-3′, light-forward: 5′-CGAGTACAAAATGAATAGCTCCG-3′, light-reverse: 5’-GCGGTTCTCCTCAATGAT-3′.

### Chromatin Sequencing

Duplicate Truseq ChIP libraries, corresponding to two biological replicates per sample, were performed by Macrogen Inc., Seoul, Korea. Sequencing was carried out using an Illumina Hiseq4000. We obtained 22–34 millions of paired-end reads for each sample, resulting in a total of 659.9 millions of paired-end reads.

### ChIPseq Visualization Patterns

ChIPseq sequenced reads were trimmed using Trimmomatic software v0.39 (Bolger et al. 2014) and aligned to the *D. buzzatii* genome downloaded from the *Drosophila buzzatii* Genome Project web page (http://dbuz.uab.cat, last accessed January 7, 2015) using Bowtie2 v2.3.5.1 (Langmead and Salzberg 2012). For the alignment, the default parameters of the *–very-sensitive-local* mode with two extra-modifications to increase the sensibility (*–local -D 20 -R 3 -L 20 -N 1 -p 12 –gbar 3 –mp 5*,*1 –rdg 4*,*2 –rfg 4*,*2*) were used to reach the highest percentage of aligned reads with both parental species and their F_1_ hybrids. Reads with map quality score lower than 30 and unmapped reads were filtered using Samtools v1.10 (Li et al. 2009) and excluded from further analysis. Deeptools v3.3.2 (Ramírez et al. 2016) was used to visualize the enrichment of each chromatin mark around the SC and the end codon (EC). First, *bamCompare* was used to normalize the ChIPseq samples by depth using the RPKM method and by the input (control). Finally, the read density values were computed using *computeMatrix* and visualized with *plotHeatmap*.

### Gene Alignments

RNAseq reads from Romero-Soriano et al. (2017) were treated the same way as ChIPseq reads to ensure that results were comparable. Gene sequences (only body region: from the SC to the EC) from *D. buzzatii* were obtained with *getfasta* of Bedtools v2.29.2 (Quinlan and Hall 2010) using the genome sequence and its gene annotations (Guillén et al. 2014). Gene sequences were masked using RepeatMasker v4.1.1. (http://www.repeatmasker.org) and the Repeat Masker from the *D. buzzatii* browser (http://dbuz.uab.cat, last accessed January 7, 2015). A total of 37 genes were completely masked and excluded from further analysis, and a total of 13,621 protein coding genes were included in the reference sequences.

To ensure that there was no bias due to the use of only the *D. buzzatii* reference genome, a de novo transcriptome for each parental species was created using Trinity v2.9 (Grabherr et al. 2011) and the corresponding RNAseq. *Drosophilabuzzatii* and *D.koepferae* transcriptomes were then aligned against all the ChIPseq inputs (supplementary file 4: Alignment_inputChIPSeq, Supplementary Material online). We also randomly selected 40 genes amongst Trinity outputs, and computed nucleotide divergence between parental species using the Mega software (Tamura et al. 2021) and the Jukes-Cantor model (Jukes and Cantor 1969) (supplementary file 4: Nucleotide_Divergence, Supplementary Material online). Both, the similar alignment percentages and the low average divergence, indicate that bias of using *D. buzzatii* as reference genome, if any, is marginal. Nevertheless, we decided to be conservative and use only the protein coding genes. Trimmed RNAseq and ChIPseq reads were aligned to the *D. buzzatii* masked gene sequences using Bowtie2 v2.3.5.1 (Langmead and Salzberg 2012) and the parameters explained above. eXpress v1.5.1 (Roberts and Pachter 2013) with the default options and then an additional online EM round (to increase the accuracy) was used to quantify read counts for each gene. All isoforms of a gene were considered together. We used rounded effective counts for the following steps.

### TE Alignment

TE RNAseq (Romero-Soriano et al. 2017) and ChIPseq read counts were analyzed using the TEcount module of the TEtools pipeline (Lerat et al. 2017) (available at https://github-com/l-modolo/TEtools). First, the manually constructed TE library containing consensus TEs from both *D. buzzatii* and *D. koepferae*, described in a previous work (Romero-Soriano et al. 2017), was aligned with the RNAseq and ChIPseq reads, using Bowtie 2 v2.2.4 (Langmead and Salzberg 2012) with the most sensitive option and keeping a single alignment for reads mapping to multiple positions (–very-sensitive). Read counts were computed per TE family, adding all reads mapped on copies from the same family. Count tables corresponding to genes and TEs were concatenated and were used for the differential expression and enrichment analyses. Genes counts were used to normalize TE counts, following the guidelines of TEtools pipeline (Lerat et al. 2017).

### Differential Expression and Enrichment Analyses

The statistical analyses were performed using R v4.0 (R Core Team 2020). The visualization of the results was performed using the R package ggplot2 v3.3.2 (Wickham 2016). The *DESeq2* function from the R Bioconductor package DESeq2 v1.28.1 (Love et al. 2014) was used to normalize read counts, using the default method, and to model the read counts using a negative binomial distribution.

For RNAseq, the DESeq2 package was used to identify differentially expressed genes and TE families while performing a Wald test (Love et al. 2014). The *P*-values were adjusted for multiple testing using the procedure of Benjamin and Hochberg (Benjamini et al. 2001) with an FDR cutoff of 0.05, and were obtained using the *results* function from the DESeq2 package. The log2FC was shrunken using the default and recommended *apeglm* algorithm (Zhu et al. 2019) of the *lfcShrink* function. Genes with an adjusted *P*-value lower than 0.05 and at least double of expression in one species above the other (absolute shrunken log2FC > 1) were considered as differentially expressed. GO term enrichment analyses of biological processes were performed for the underexpressed and overexpressed significant genes using the topGO R package v.2.42.0. (Alexa and Rahnenfuhrer 2020) (“weight01” algorithm and Fisher’s statistic) and the ortholog genes in *D. melanogaster* obtained from the *D.buzzatii* Genome Project web page (http://dbuz.uab.cat, last accessed January 7, 2015). The obtained Fisher’s exact test *P*-values were not adjusted but, from the total gene ontologies with *P* < 0.05, only the top $\frac{1}{3}$ with the lowest *P*-values were considered as enriched.

Regarding the ChIPseq data, a handmade script was used to perform the differential enrichment analysis between hybrids and both parental species. Briefly, the “Regularized log” transformed counts obtained using DESeq2 for each chromatin mark were considered and analyzed using the following linear model for each gene: RldKm ∼ RldKi + species (RldKm: log-transformed counts for the considered histone mark, RldKi: log-transformed counts for the input). We considered contrasts between pairs of species modalities (*D. buzzatii*, *D. koepferae*, hybrid). Because of the high number of tests and the lack of extreme *P*-values, the Benjamin and Hochberg (Benjamini et al. 2001) adjustment of the *P*-values had a high effect in our results. For this reason, the *P*-values were not adjusted and, from the genes and TE families with *P* < 0.05, the rounded $\frac{1}{3}$ of the top genes and TE families with the lowest *P*-values were considered as differentially enriched.

The significant genes were assigned to their respective chromosomes following the scaffold assignation to chromosomes obtained from a previous work (Guillén et al. 2014) to detect potential chromosome biases of differentially expressed genes. Additionally, the order of the significant TE families was analyzed to detect possible order biases in our significant TEs.

Within each genome, we quantified the contribution of histone mark enrichments on transcript amounts using the following linear model on log-transformed read counts for each gene and TE family: RNA ∼ K4 + K9 + K27 + Input. In addition, we tested whether changes in RNAseq counts in hybrids were associated with changes in ChIPseq counts using Fisher’s exact test on 2 × 2 matrices: genes and TE families were classified as displaying a positive or negative log2FC of RNAseq counts in hybrids versus parents and as displaying an increase or decrease in histone mark enrichment in hybrids versus parents.

### Allele-Specific Expression Analysis

We created a de novo transcriptome with the RNAseq of both parental species, together with the hybrids using Trinity v2.9 (Grabherr et al. 2011). A SuperTranscript (Davidson et al. 2017) was then created as a general reference transcriptome. The GATK pipeline for variant calling (Van der Auwera and O’Connor 2020) was used to detect differences between the general reference transcriptome and each parental species. The VCF files were filtered following the GATK guidelines, including a coverage depth of at least 10, and the exclusion of variants only present in one replicate, which were considered assembly errors. These variants were replaced in the general reference transcriptome using the FastaAlternateReferenceMaker GATK tool (Van der Auwera and O’Connor 2020) to create a reference transcriptome for each parental species.

HISAT2 (Kim et al. 2015) with the “no-softclip” option was used to align the RNAseq from hybrids to both parental reference transcriptome. Then, CompareBams (Lindenbaum 2015) of Jvarkit was used to compare the alignments and FilterSamReads from Picard (Broad Institute 2018) to filter out the reads aligning in different position when the data were aligned to each parental species. Samtools v1.10 (Li et al. 2009) was used to remove multimapped and unmapped reads and BamTools v.2.4.0 (Barnett et al. 2011) to keep only reads that align without mismatches. Bedtools multicov (Quinlan and Hall 2010) and a manually updated GTF were used to count reads aligning to each gene.

The reference transcriptomes were annotated using BLAT v.35x1 (Kent 2002) with the parameters –minIdentity = 80 and –maxIntron = 75,000 and the gene sequences of *D. buzzatii*, keeping the best match with an overlap of at least 50%. Subsequently, statistical analyses were performed using R v4.0 (R Core Team 2020) and DESeq2. The *DESeq2* function was used to normalize read counts, using the default method. First, the *results* function was used to compute the log2FC and the adjusted *P*-values using the Benjamin and Hochberg (Benjamini et al. 2001) method. A FDR cutoff of 0.05 was used to identify differentially expressed genes between parental species (P). Second, the same procedure and cutoff was used to identify genes with a different abundance of the parental and the maternal allele in hybrids (H), and were considered as genes with *cis-*regulatory divergence. Finally, significant genes in either P or H were analyzed for *trans-*regulatory effects (T) by comparing the P and H ratios with the same *results* function. Genes were then categorized in the following groups as reported in a previous work (McManus et al. 2010):

- *Cis-* only: Significant differential expression in P and H, but no significant T.
- *Trans-* only: Significant differential expression in P, and T, but not H.
- *Cis-* + *trans-*: Significant differential expression in P, H, and T. *Cis-* and *trans-* regulatory differences favor expression of the same allele.
- *Cis-* × *trans-*: Significant differential expression in P, H, and T. *Cis-* and *trans-* regulatory differences favor expression of opposite alleles.
- Compensatory: Significant differential expression in H, and T, but not P. *Cis-* and *trans-*regulatory differences compensate each other, resulting in no expression differences between parental species.
- Conserved: No significant differential expression in H, P, or T. Conserved regulation.
- Ambiguous: Other patterns with no clear biological interpretation.

### Piwi Pathway Genes and piRNA Amount Study

Additionally, reciprocal tblast v2.10.1. (Camacho et al. 2009) of the *D.melanogaster* piRNA pathway proteins from UniProt (The UniProt Consortium 2021) and the reference gene sequence list were performed. A total of 31 proteins were detected and associated with *D. buzzatti* genes. The *Argonaute 3* gene (*ago3*), not included in the reference genes, was manually included in the list from our previous results (Gámez-Visairas et al. 2020).

Small RNAseq raw reads from our previous work (Romero-Soriano et al. 2017) were used to study the piRNA regulatory data in the TE expression study. Using *PRINSEQ lite* (Schmieder and Edwards 2011), we isolated 23–30 nt-long reads and considered them as piRNAs. For normalization purposes, we also isolated 20–23 nt-long reads and searched for microRNA sequences: low-quality reads were removed using UrQt (Modolo and Lerat 2015), then trimmed 20–23 nt reads were aligned to the masked genes using Bowtie V.1.3.0 (Langmead et al. 2009) and keeping a single alignment for reads mapping to multiple positions. These reads were considered to correspond to microRNAs and counts were computed using eXpress v1.5.1 (Roberts and Pachter 2013). TE counts among piRNAs were computed using the TEcount module of TEtools (Lerat et al. 2017). piRNA counts were then normalized so that the sum of microRNA counts is the constant across samples.

Regarding TE sequences, RNAseq and CHIPseq data were integrated following the same approaches and processes as for genes. To get insight into the chromatin mark combination, we study the association between the euchromatic mark H3K4me3 and the heterochromatic marks (H3K9me3 and H3K27me3) in hybrids versus each parental species.

### Statistical Tests

Three main statistical tests were used in the article and were performed using the R v4.0 (R Core Team 2020) program:

- *Two proportion Z-test* was used to compare distributions of significant sequences (genes and TE families) across comparisons and *cis-* and *trans-*regulatory classes.
- *Chi-square test* under equal assumption was used to detect chromosome-biases and TE-category-biases.
- *Fisher’s exact test* under independence assumption was computed using a 2 × 2 contingency table to detect associations in: gene and TE expression (log2FC) and chromatin mark enrichment (size effect); TE euchromatic mark (H3Kme3) and heterochromatic marks (H3K9me3 and H3K27me3); *trans-* and *cis-*regulatory divergence and differences of expression between parental species; compensatory and remaining classes and gene deregulation in hybrids, and TE expression and piRNA quantities, in hybrids versus each parental species independently.

The results were corrected for multiple testing using the Benjamin and Hochberg (Benjamini et al. 2001) method.

## Supplementary Material

Supplementary data are available at *Genome Biology and Evolution* online.

## Supplementary Material

evac024_Supplementary_Data
