# Supplementary material for: High Stability of the Epigenome in Drosophila Interspecific Hybrids
Source: Genome Biol Evol. 2022 Feb 10;14(2):evac024. doi: 10.1093/gbe/evac024 (PMC8872975; doi:10.1093/gbe/evac024)
Supplement: evac024_Supplementary_Data [file evac024_supplementary_data.zip › Supplementary file 3.pdf]

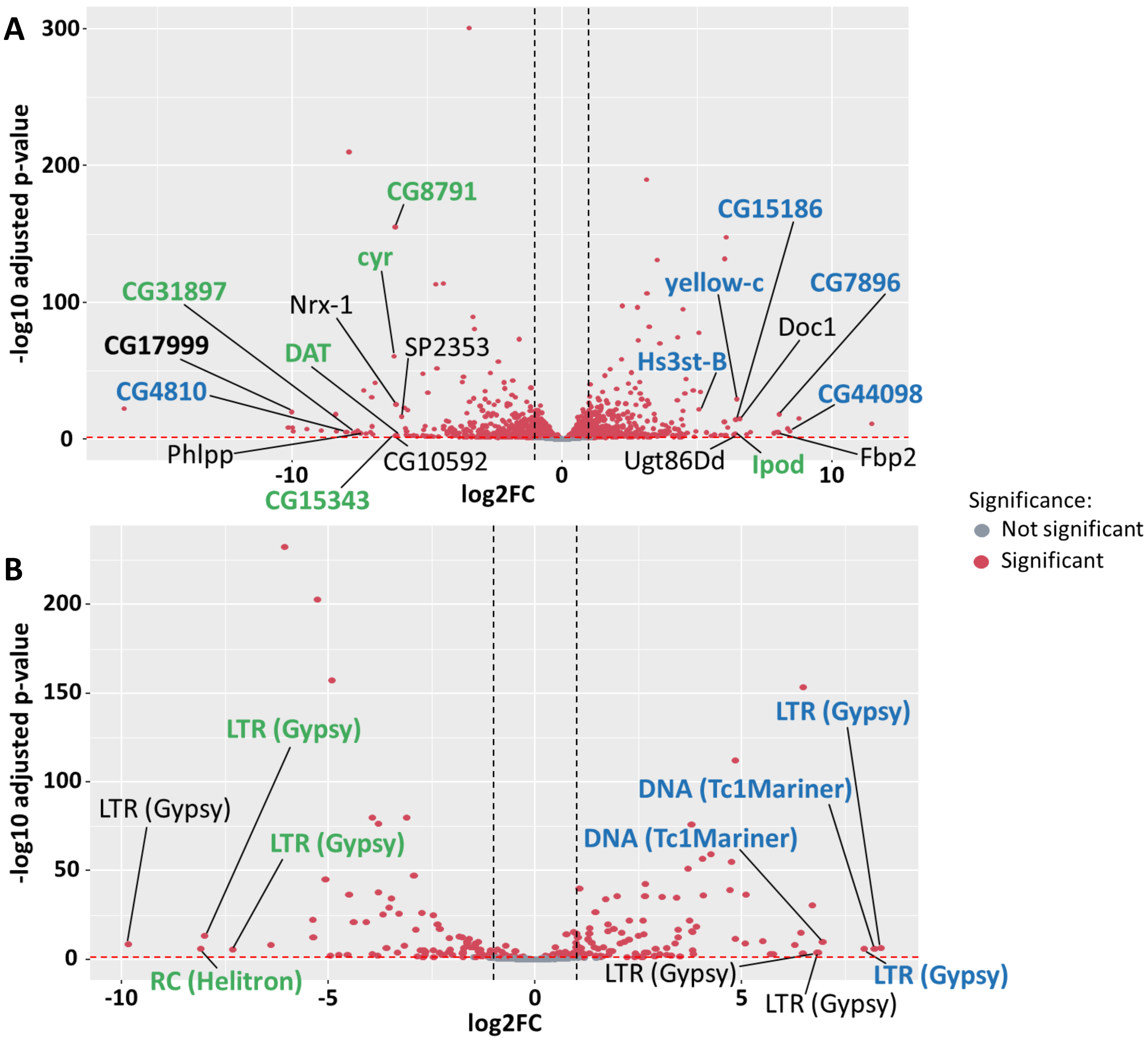

Figure S1. Differential gene (**a**) and TE family (**b**) expression analyses in *D. buzzatii vs D. koepferae*. Positive log2FC values correspond to genes (**a**) and TE families (**b**) more expressed in *D. buzzatii*. The genes showing the 20 highest log2FC values and displaying an ortholog in *D. melanogaster* are shown in **a**. The TE superfamilies/orders showing the 10 highest log2F values are shown in **b.** Genes (**a**) and TE families (**b**) common to the examples in the hybrids *vs D. buzzatii* comparison are shown in green, hybrids *vs D. koepferae* are shown in blue and in all three comparisons are in bold.

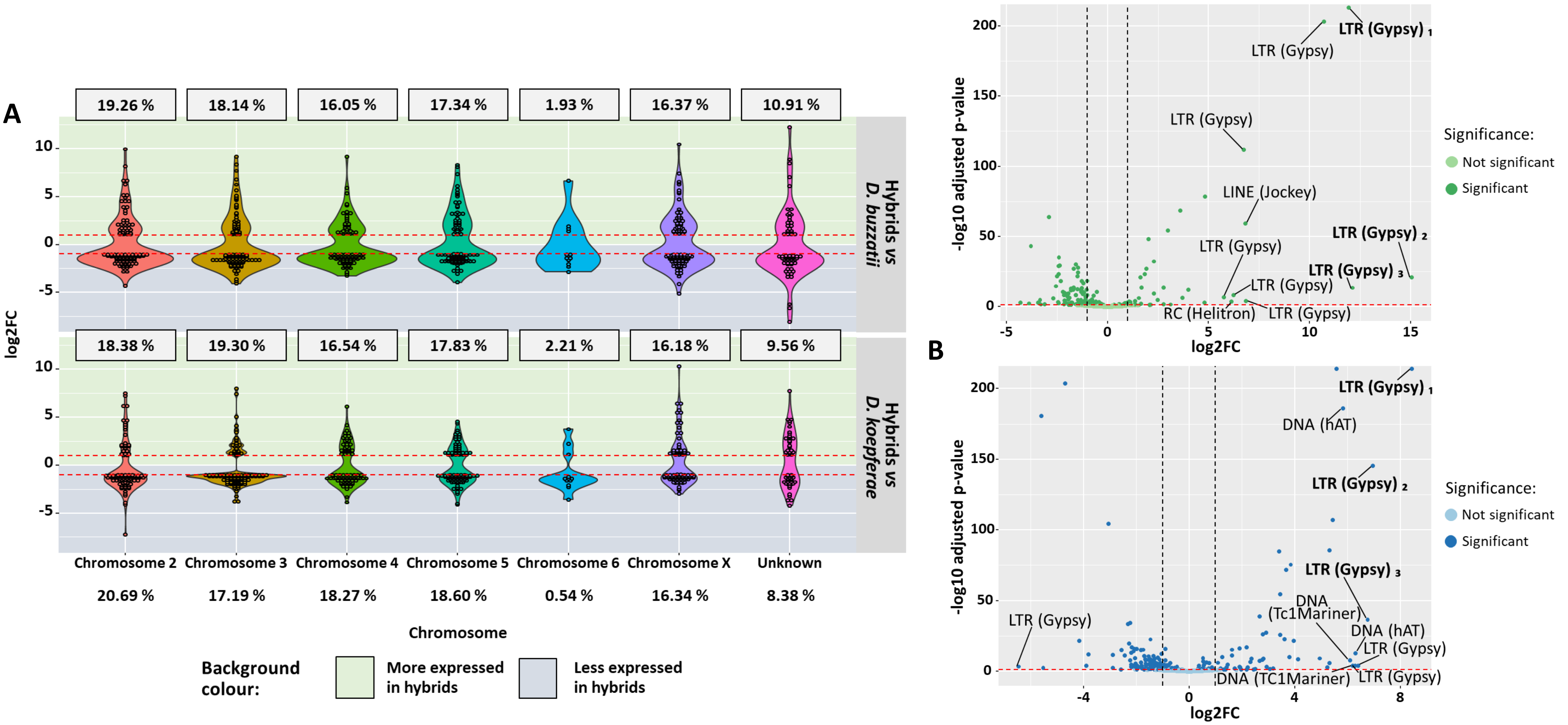

Figure S2. a Violin plots representing the chromosomal distribution of differentially expressed genes in hybrids *vs* parental species. Genes with an unknown location were categorized as unknown. Points indicate the log2FC of each gene. The percentages of differentially expressed genes per chromosome are framed. The expected (total) gene percentages are at the bottom. Red-dashed lines indicate the log2FC threshold (±1). **b** Differential TE family expression analyses in hybrids *vs* *D. buzzatii* (green) and *D. koepferae* (blue). Positive log2FC values correspond to TE families more expressed in hybrids. The TE superfamilies/orders showing the 10 highest log2FC values are shown. TE families common to both comparisons are in bold and identified with a subscript.

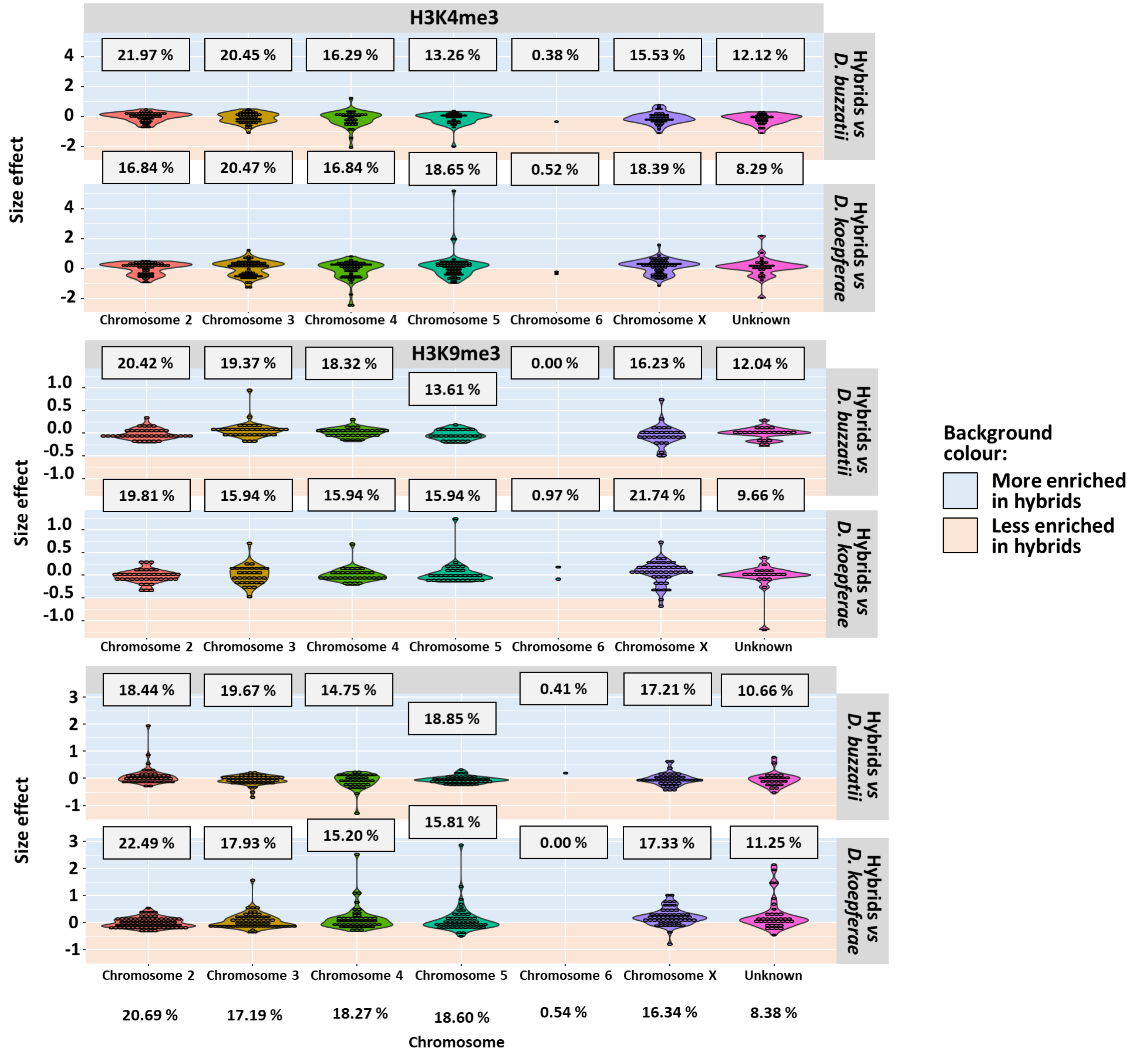

Figure S3. Violin plots representing the chromosomal distribution of differentially H3K4me3, H3K9me3 and H3K27me3 enriched genes in hybrids *vs* parental species. Points indicate the size effect. The percentages of differentially enriched genes per chromosome are framed. The expected (total) gene percentages per chromosome are at the bottom.

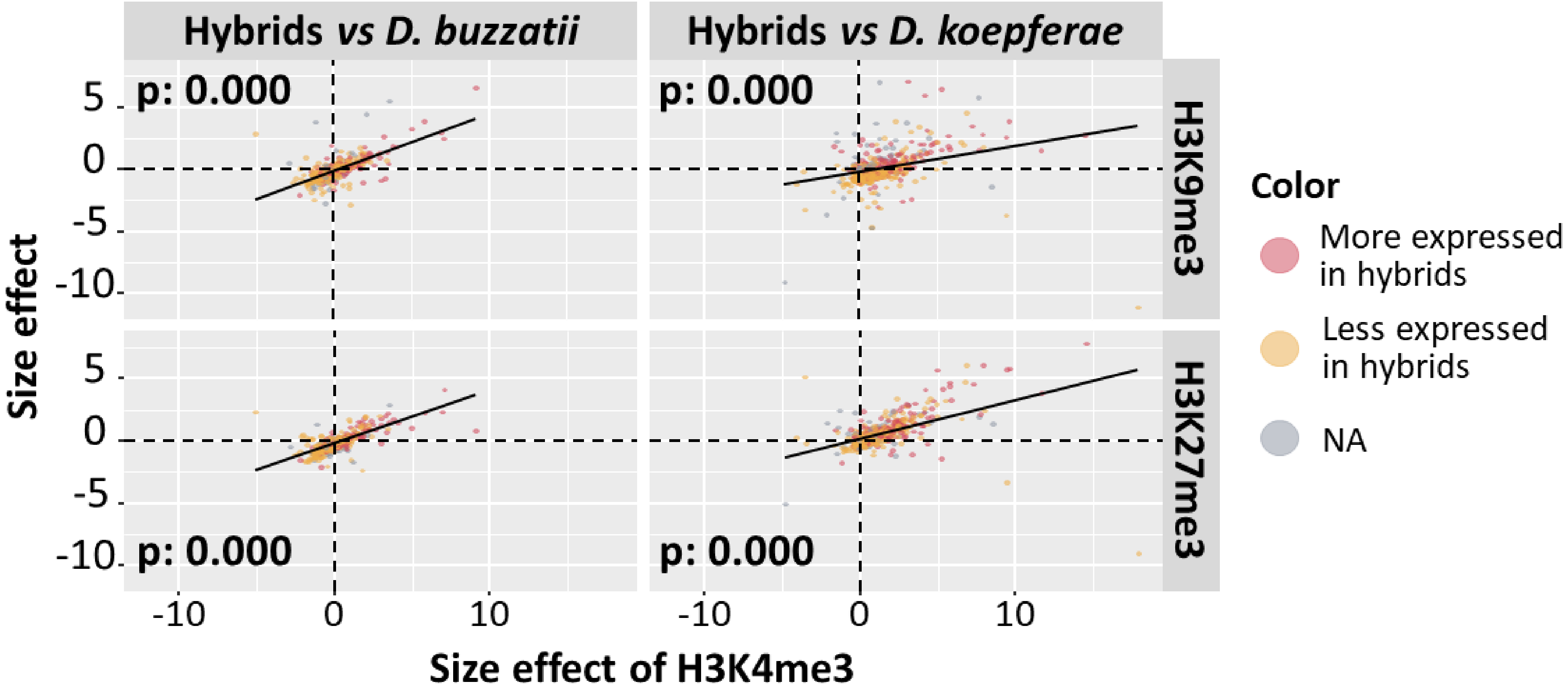

Figure S4. Association between H3K4me3-H3K9me3 and between H3K4me3-H3K27me3 enrichment in hybrids *vs D. buzzatii* and *D. koepferae* respectively. Colours represent TE families differential expression in hybrids *vs* parental species. Linear model p-values are shown in the corner.

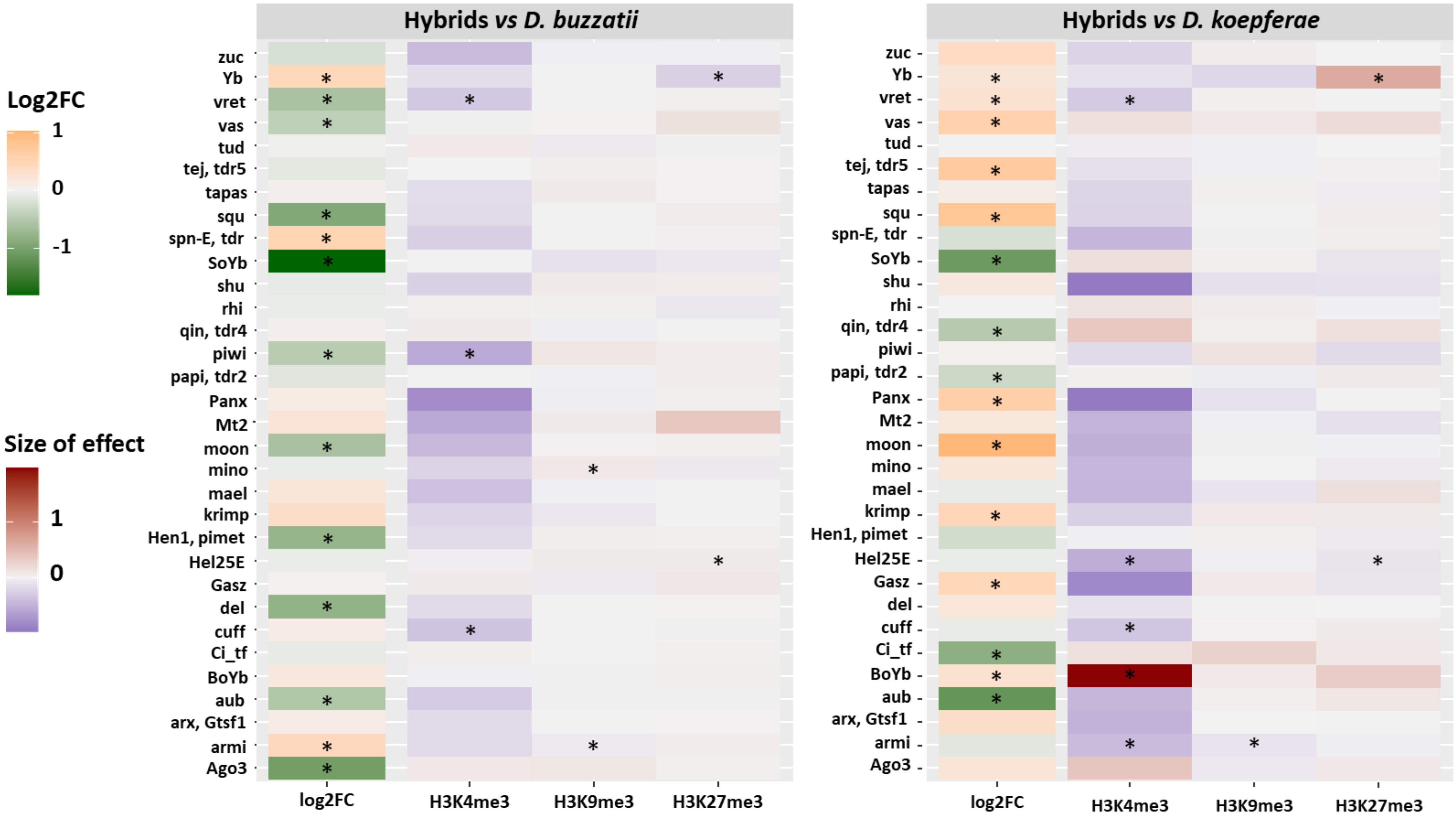

Figure S5. Differential expression (log2FC) and enrichment analyses (size effect) of piRNA Pathway Genes in hybrids *vs* parental species. Significant values ($p < 0.05$) are indicated with an asterisk (*). Colours indicate the values of the differences in gene expression (green - orange) and enrichment (purple - red).
